# A Modern "Nightstick Fracture" Induced by Contemporary Ballistics During the George Floyd Protests

**DOI:** 10.7759/cureus.13530

**Published:** 2021-02-24

**Authors:** Braden J Passias, Benjamin C Taylor, Devon Myers, John Casey

**Affiliations:** 1 Orthopedic Surgery, OhioHealth Doctors Hospital, Columbus, USA; 2 Orthopedic Trauma, OhioHealth Grant Medical Center, Columbus, USA; 3 Emergency Medicine, OhioHealth Doctors Hospital, Columbus, USA

**Keywords:** ulnar fracture, nightstick fracture, riot control, ballistics

## Abstract

A 28-year-old male presented to the emergency department with an isolated ulnar shaft fracture secondary to a ballistic injury with a wooden pellet gun. This injury is also known as a “nightstick fracture,” which is a common eponym in orthopedic surgery used to describe a fracture of the ulnar shaft. The eponym gained its title for the injury commonly seen when in a defensive position while being attacked with a wooden club. It is widely accepted that this infamous injury was popularized in the 1960s as a sequela of the many race-related riots across the United States.

This case details how the nightstick fracture is still prevalent as a result of political protesting today, despite modern-day methods of non-lethal riot control.

## Introduction

The “nightstick fracture” is a common eponym in orthopedic surgery used to describe a fracture of the ulnar shaft. The injury is termed for a fracture resulting from a defensive position when being struck with a police baton, causing an isolated fracture of the ulnar diaphysis [1]. Although it is unknown when this eponym was first established within the medical community, it is widely accepted that this mechanism of injury was popularized in the 1960s as a sequela of the many race-related riots across the United States [2]. Nearly 60 years later, we see the same clinical diagnosis secondary to analogous political unrest; however, this case was induced by a modern ballistic cannon as opposed to an antiquated wooden truncheon.

## Case presentation

**Figure 1:**
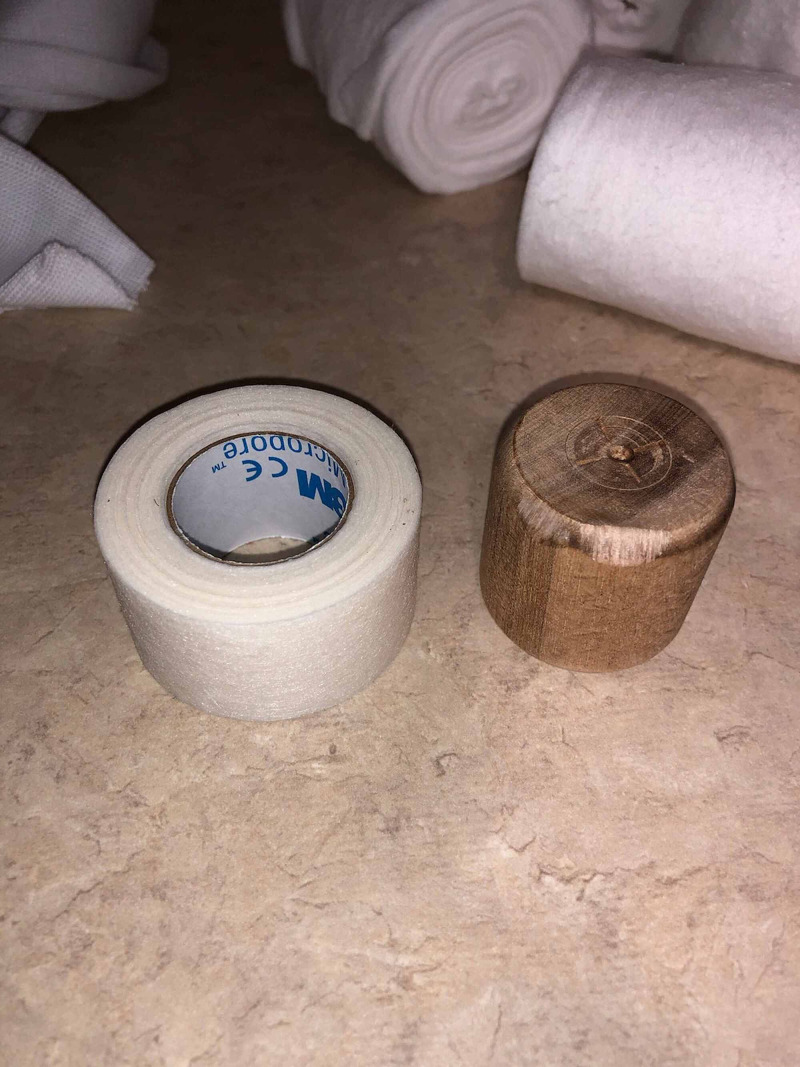
Wooden projectile fired from a police cannon as part of non-lethal riot control measures compared to a roll of tape (for size comparison) to adequately demonstrate dimensions.

A 28-year-old male presented to the emergency department with left forearm pain following a riot-related ballistic injury 12 hours prior. The patient sustained the injury while engaged in an urban protest in light of the recent controversial death of Minneapolis resident George Floyd. As a part of non-lethal measures of riot control, the patient was hit in the arm with a wooden projectile fired from a police officer cannon (Figure 1).

**Figure 2:**
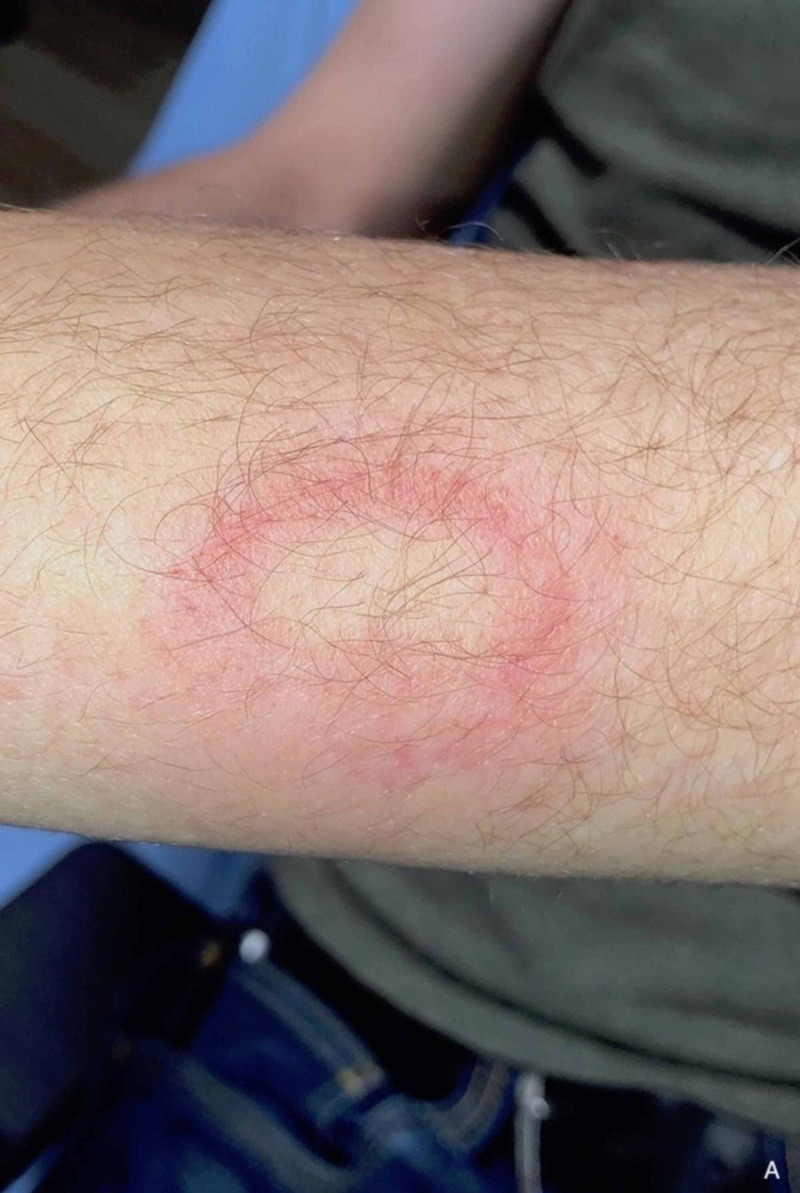
Clinical photograph depicting a forearm wound from the wooden projectile.

**Figure 3:**
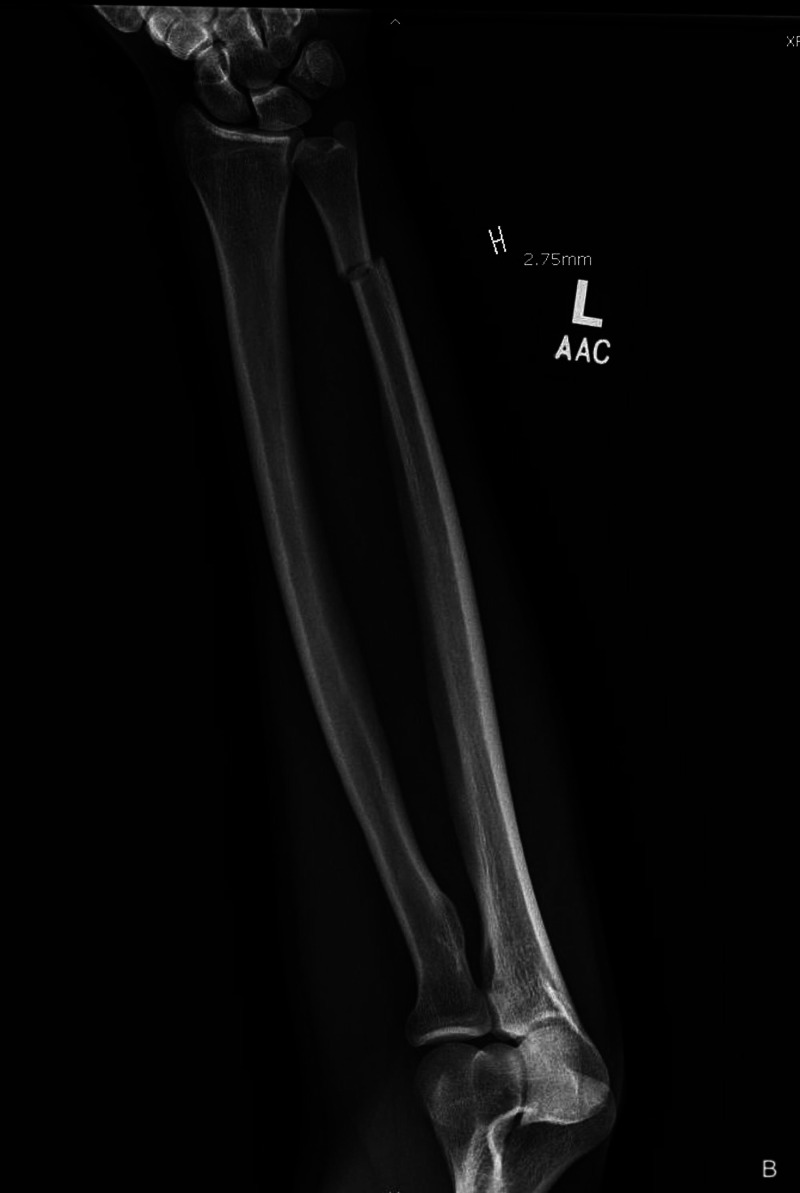
Anteroposterior injury film/radiograph of the left forearm demonstrating an isolated ulnar shaft fracture with minimal displacement and angulation.

**Figure 4:**
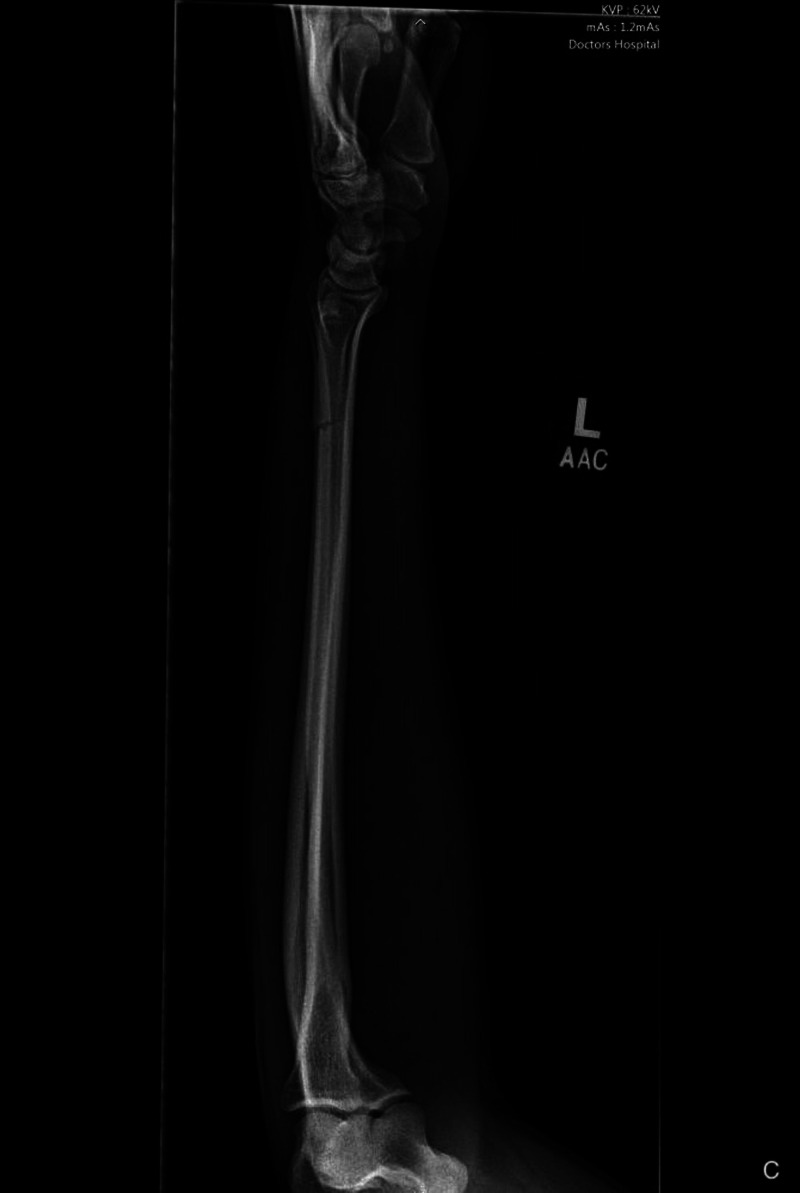
Lateral injury film/radiograph of the left forearm demonstrating an isolated ulnar shaft fracture with minimal displacement and angulation.

Standard clinical and radiographic workup determined that the patient had an isolated closed fracture of the ulnar diaphysis, more commonly known by its eponym, a “nightstick fracture” (Figures 2-4). After ruling out any excessive compartmental pressure with clinical examination, the patient was determined to be in absence of any distal radioulnar joint or radial head instability that could have resulted secondary to this high energy injury. Fracture displacement and angulation were well within the acceptable tolerances, and the patient was placed in a short arm cast in neutral pronation/supination for a six-week immobilization period. Informed consent for participation in clinical research endeavors was obtained prior to the initiation of this publication.

## Discussion

**Figure 5:**
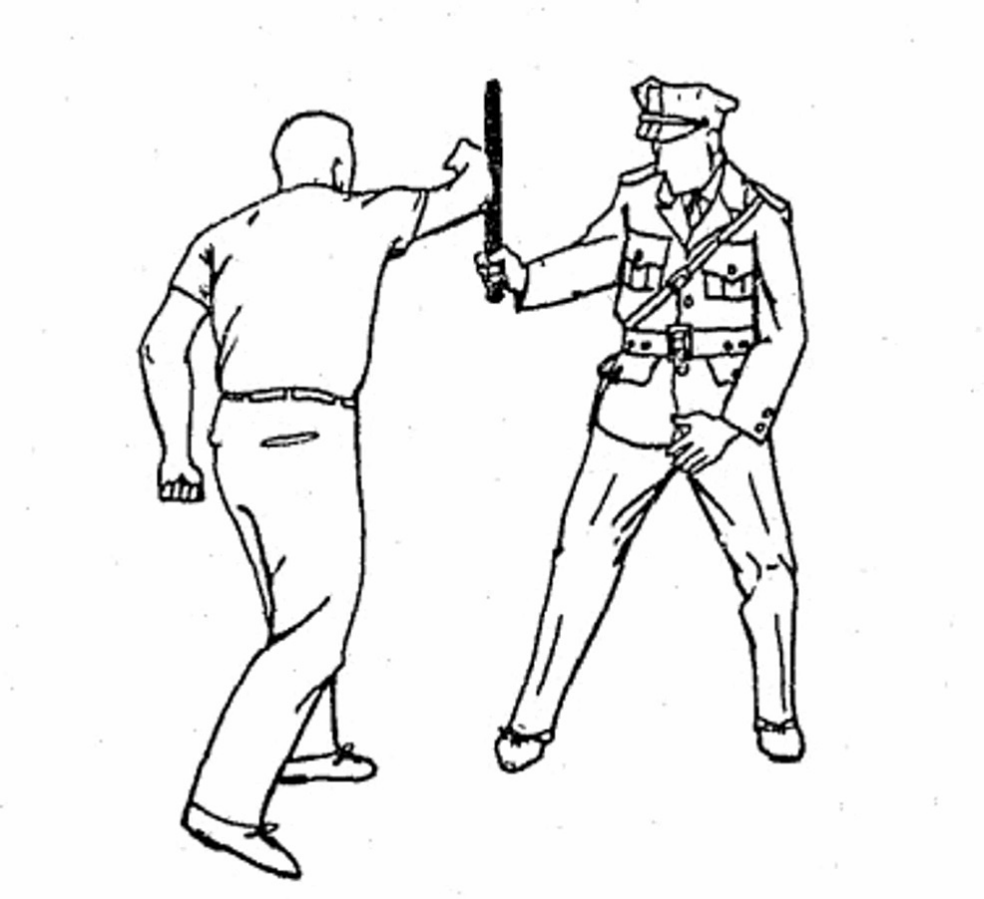
"Nightstick" Injury Mechanism Pictorial sketch adopted from a 1967 police handbook issued by the Federal Bureau of Investigation detailing how to use a wooden baton as a non-lethal measure for riot control.

The wooden baton has an elaborate history as a non-lethal weapon in the police force. This wooden club eventually became a symbol for police officers worldwide, but it was first commercialized overseas in London, England, during the Victorian era [3]. London’s first police department was founded in 1829 by Prime Minister Sir Robert Peel, and he dictated that police officers would be unarmed except for that of a wooden club [3]. As an effort to modernize the police force, this was Peel’s attempt to “police by consent” - as opposed to intimidation - and to hopefully gain the respect of the public during times of political unrest. Over time, and as this weapon became more popular in the police force in the United States, the wooden baton adopted multiple other titles, including the “nightstick” (Figure 5). Based on a size larger than that of a “billy club” and smaller than that of a “riot stick,” the nightstick was the most common type of baton carried by officers “on the beat,” as described by a 1967 police handbook issued by the Federal Bureau of Investigation detailing how to use a wooden baton as a non-lethal measure for riot control [4].

Participating in an active protest related to racial segregation, this patient suffered the same injury that was popularized in the “Long hot Summer of 1967,” when police officers used batons as the primary weapon to patrol protesters [2]. Most commonly known for the uprisings in Detroit and Newark, there were nearly 160 riots across the United States brought on by similar frustrations with racial inequality. Sixty years later, we see the same orthopedic pathology secondary to comparable political turmoil; however, this patient’s injury was induced by modern riot control weapons.

The subcutaneous location of the ulna renders it susceptible to injury when exposed to blunt trauma. Furthermore, a biomechanical study investigating the area moment of inertia in forearm osteology found that the junction of the middle and distal third of the ulnar shaft region may have increased vulnerability to fracture due to a lower cortical density [5]. Given the subcutaneous location and a predisposition for cortical disruption, the ulnar diaphysis was ripe for fracture after this patient was hit with a “broomstick round,” a modern non-lethal weapon of riot control [6].

In addition to proper radiographic workup, isolated ulnar shaft fractures warrant further considerations of instability on clinical exam. Using the “shuck test,” or imaging the contralateral wrist as a comparison film, the examiner can rule out any instability of the distal radioulnar joint. Ranging the elbow to evaluate for any radial head pathology is also paramount, as several investigations have shown that isolated ulnar shaft fractures could actually be Monteggia fractures that have spontaneously reduced [7,8]. As with other extremity fractures, a thorough neurovascular exam should be given to exclude any early manifestations of compartment syndrome.

Traditionally, non-operative treatment was mainly recommended for non-displaced fractures or fractures with less than 50% displacement [9-12]. The biomechanical analysis demonstrates that more than 50% displacement involves considerable disruption of the periosteum and of the interosseous membrane, rendering the fracture highly unstable and thus warrants operative management [13]. Non-operative management of ulnar shaft fractures traditionally involves a period of short arm cast immobilization with close radiographic follow up. Previous investigations have suggested that immobilization of the forearm in neutral, supination, or pronation positions has been based on theory, anecdotal experience, and tradition [14,15]. Anecdotally, a common practice in our system warrants casting in a mid-prone position.

## Conclusions

In conclusion, the ulnar shaft fracture, more commonly known as the “nightstick fracture,” is still prevalent today as an orthopedic injury secondary to political protests, despite modern-day improvements in non-lethal riot control weapons.
